# A Rare Geriatric Diagnosis of Eosinophilic Granulomatosis With Polyangiitis: A Case Report

**DOI:** 10.7759/cureus.35398

**Published:** 2023-02-24

**Authors:** Fábio C Emidio, Rafaela C Pereira, Frederico R Martins, Roberto C Marques, Teresa Martins

**Affiliations:** 1 Internal Medicine Department, Centro Hospitalar Universitário do Algarve - Hospital de Faro, Faro, PRT; 2 Rheumatology Department, Centro Hospitalar Universitário do Algarve - Hospital de Faro, Faro, PRT; 3 Nephrology Department, Centro Hospitalar Universitário do Algarve - Hospital de Faro, Faro, PRT

**Keywords:** churg-strauss syndrome, pulmonary eosinophilia, geriatric, allergic granulomatous angiitis, eosinophilic granulomatous vasculitis

## Abstract

Eosinophilic granulomatosis with polyangiitis (EGPA) is a rare form of systemic disease characterized by inflammation and necrotizing effects of the small and medium blood vessels. It is a vasculitis found in all age groups and both genders, although its etiology is unknown. The mean age at diagnosis is 40 years, consisting of an uncommon cause of vasculitis in people older than 65 years. It is the least common of the three antineutrophil cytoplasmic antibody (ANCA)-associated vasculitis (EGPA, granulomatosis with polyangiitis (GPA), and microscopic polyangiitis). The characteristic features of EGPA include extravascular eosinophilic granulomas, peripheral eosinophilia, and asthma, usually responsive to steroid treatment.

In this article, we discuss a case of an 83-year-old male with a history of undetermined etiology of chronic kidney disease, chronic obstructive pulmonary disease (COPD), and severe chronic rhinosinusitis with nasal polyposis. First hospitalized with the suspicion of community-acquired pneumonia (CAP), based on worsening blood eosinophilia and unresolving respiratory symptoms, a suspicion for EGPA was raised. The development of an eosinophilic pleural effusion, later upon admission, was a predominant factor for its confirmation, as it constitutes a rare finding, only present in about 30% of patients. Laboratory tests showed elevated IgE, the presence of antineutrophil cytoplasmic antibodies directed against myeloperoxidase with a perinuclear staining pattern (ANCA-MPO), and the absence of antiproteinase 3 (anti-PR3) ANCA, which were consistent with the diagnosis. A pleural biopsy was then made, revealing fibrosis with the presence of eosinophils, although with no evidence of granulomas. According to the most recent and accepted classification criteria, the “2022 American College of Rheumatology and European Alliance of Associations for Rheumatology (ACR/EULAR) for EGPA,” this patient presented with a score of 13 (a score greater than or equal to 6 is needed for the classification of EGPA). Hence, a diagnosis of EGPA was assumed, and the patient was initiated on corticosteroid therapy, with a favorable response.

The aim of this article is to present a rare case of EGPA diagnosis made at the age of 83 years old, although there was evidence that could point to this disease years before the diagnosis was made. In the present case, it is important to point out the long diagnostic delay in a geriatric patient, much older than the median age of diagnosis for EGPA, culminating in a curious case of uncommon pleuroparenchymal involvement.

## Introduction

Eosinophilic granulomatosis with polyangiitis (EGPA) is a rare form of systemic disease characterized by inflammation and necrotizing effects of the small and medium blood vessels. It is a vasculitis found in all age groups and both genders, although its etiology is unknown [1]. The mean age at diagnosis is 40 years, consisting of an uncommon cause of vasculitis in people older than 65 years, accounting for 5% of histologically proven vasculitis among 38 older adult patients with various systemic forms of angiitis [2-4]. First described by Churg and Strauss in 1951 (hence the currently in disuse term Churg-Strauss syndrome), it is associated with extravascular eosinophilic granulomas, peripheral eosinophilia, and asthma. It is estimated that 10% of patients with a major form of vasculitis are recognized to have EGPA, being the least common of the three anti-neutrophil cytoplasmic antibody (ANCA)-associated vasculitis (EGPA, granulomatosis with polyangiitis (GPA), and microscopic polyangiitis) [5].

The prevalence of EGPA in Europe ranges from 10.7 to 14 million, and in the United States, it is approximately 18 million [6]. The most actual and accepted classification criteria were defined by the “2022 American College of Rheumatology and European Alliance of Associations for Rheumatology (ACR/EULAR) for EGPA” [7]. These include clinical criteria (obstructive airway disease, nasal polyps, and mononeuritis multiplex) and criteria obtained from laboratory and biopsy results (blood eosinophil count greater than or equal to 1 × 10^9^, extravascular eosinophilic-predominant inflammation on biopsy, a positive test for cytoplasmic antineutrophil cytoplasmic antibodies (cANCAs) or antiproteinase 3 (anti-PR3) antibodies or hematuria) [7]. A score greater than or equal to 6 is needed for classification. Prognosis has improved significantly since the widespread use of systemic glucocorticoids and the selected use of immunosuppressant agents for patients with more severe diseases. Patients tend to respond well to steroid treatment, although follow-up is characterized by frequent relapsing [1]. The presence or absence of the features that make up the Five Factor Score (FFS) has been used to predict survival in EGPA (age > 65 years, cardiac insufficiency, renal insufficiency (stabilized peak creatinine: 1.7 mg/dL (150 micromol/L)), gastrointestinal involvement, and absence of ear, nose, and throat (ENT) manifestations). Each of these factors is given one point. The FFS ranges from 0 to 2: a score of 0 is given when none of the factors is present, a score of 1 for one factor, and a score of 2 for two or more factors [8]. One case series of 118 patients followed for six years found a mortality rate of 14% among 44 patients with an FFS ≥ 1 and an overall mortality of 10% [9].

The aim of this article is to present a rare case of EGPA diagnosis made at the age of 83 years old, although there was evidence that could point to this disease years before the diagnosis was made.

## Case presentation

We present the case of an 83-year-old male with a medical history of chronic kidney disease under hemodialysis, bilateral ureterostomy and radical cystectomy for bladder cancer, chronic obstructive pulmonary disease (COPD), severe chronic rhinosinusitis with nasal polyposis, and cardiac diastolic dysfunction. The patient was admitted to the emergency department with progressive dyspnea and productive cough of three days duration, without fever or other constitutional symptoms.

**Figure 1:**
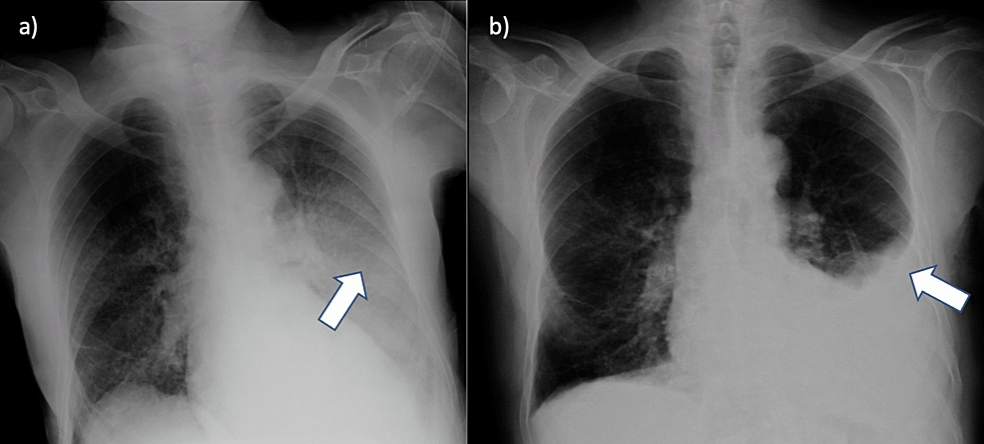
Chest X-ray a: Upon admission, note a diffuse opacity in the left middle and lower lung fields (arrow). b: On the 11th day, a pleural effusion is clearly visible in the lower third of the left lung field (arrow).

He was hemodynamically stable (heart rate (HR): 82 bpm, blood pressure (BP): 145/89 mmHg). At physical examination, he was in respiratory distress (respiratory rate (RR): 22 rpm), with abolished lung sounds at the left medium/lower field upon auscultation. Arterial gasometry showed mild type 1 respiratory insufficiency (partial pressure of oxygen (PO2): 54.6 mmHg, tissue (muscle) oxygen saturation (StO2): 87%), and laboratory results revealed leukocytosis (12.3 × 10^9^/L), neutrophilia (79%), mild eosinophilia (0.5 × 10^9^/L, 4.4%), and elevated C-reactive protein (135 mg/L) (normal range: <5 mg/L). The thoracic radiography revealed a diffuse opacity in the left middle and lower lung fields. He was initially admitted to the medicine department with the presumptive diagnosis of community-acquired pneumonia (CAP) and was started with antibiotic therapy with levofloxacin (allergy to penicillin) IV and supplemental oxygen (nasal cannula with 3 L/minute). During the first 11 days, the patient remained without favorable clinical, laboratory, or imaging response, despite broadening antibiotic coverage to gentamicin plus vancomycin on the eighth day. On the 11th day of admission, the patient remained dependent on supplemental oxygen (SpO2 90% at 3.5 L/minute with nasal cannula, pO2: 74 mmHg), and radiographic reevaluation revealed a pleural effusion in the lower third/base of the left lung, previously not present upon admission (Figure 1).

Laboratory tests showed progressive peripheral eosinophilia (1 × 10^9^/L, 14%). Thoracentesis (evacuative) revealed a clear, citrine yellow exudative pleural effusion (pH: 7.5, total protein: 3, LDH: 146 IU/L, cell count: 852 leucocytes (56% polymorphonuclears, 26% neutrophils, and 30% eosinophils), negative culture results including mycobacteria, pleural fluid LDH/serum LDH ratio: 0.76, and pleural fluid protein/serum protein ratio: 0.54). Immunology tests revealed the following: rheumatoid factor < 8.9 UI/mL (normal range: <20 UI/mL), serum immunoglobulin E (IgE): 684 UI/mL (normal range: <88UI/mL), negative antinuclear antibodies (ANAs), positive antineutrophil cytoplasmic antibodies (ANCA) directed against myeloperoxidase with a perinuclear staining pattern (MPO-ANCA) (titer: >300 UA/mL) (normal range: <15 UA/mL), and negative anti-PR3-ANCA (titer: 2 UA/mL) (normal range: <15 UA/mL). The presence of both peripheral and pleural eosinophilia raised suspicion for EGPA. Therefore, a pleural biopsy was done, revealing an inflammatory lymphocytic infiltrate with eosinophils and fibrosis, although with no reference to granulomas. EGPA was the assumed diagnosis, and the patient was started on corticosteroid treatment with prednisolone 1 mg/kg/day (50 mg), which resulted in a rapidly favorable response, progressive weaning of supplemental oxygen, and resolution of the pleural effusion.

## Discussion

In this case, based on worsening blood eosinophilia and unresolving respiratory symptoms, a suspicion for EGPA was raised. The development of an eosinophilic pleural effusion, later upon admission, was a predominant factor for its confirmation, as it constitutes a rare finding, only present in about 30% of patients [10]. Noteworthy is also the previous history of pansinusitis and severe chronic rhinosinusitis with nasal polyposis, well established as symptoms of the disease but never identified as such. The patient presented as well with airway involvement diagnosed as a chronic obstructive pulmonary disease (COPD) group D, with spirometry in 2015 describing a severe obstructive type ventilatory alteration, which did not show significant reversibility after the administration of a bronchodilator and severe decrease in diffusing capacity for carbon monoxide (DLCO) compared to theoretical values. According to the classification criteria defined by the “2022 ACR/EULAR for EGPA” [7], this patient presented with obstructive airway disease (+3 points), nasal polyps (+3 points), blood eosinophil count of 1 × 10^9^ that developed later at the hospital (+5 points), extravascular eosinophilic-predominant inflammation on biopsy (+2 points), and a negative test for cytoplasmic antineutrophil cytoplasmic antibodies (cANCAs) or anti-PR3 antibodies (+3 points), resulting in a total score of 13 points; therefore, this case falls under the classification of EGPA (Table 1).

**Table 1:** 2022 classification criteria for EGPA Defined by the “2022 American College of Rheumatology and European Alliance of Associations for Rheumatology (ACR/EULAR) for EGPA” [7]

Clinical criteria	Points
Obstructive airway disease	+3
Nasal polyps	+3
Mononeuritis multiplex	+1
Laboratory and biopsy criteria	Points
Blood eosinophil count > 1 × 10^9^/L	+5
Extravascular eosinophilic-predominant inflammation on biopsy	+2
Positive test for cytoplasmic antineutrophil cytoplasmic antibodies (cANCAs) or antiproteinase 3 (anti-PR3) antibodies	-3
Hematuria	-1

In this classification, negative items were included because these are intended to be used as classification criteria, not as diagnostic criteria. This distinction is important: the main objective is to differentiate EGPA from other forms of vasculitis. Therefore, the criteria for hematuria and anti-PR3-ANCA punctuate as negative items as anti-PR3-ANCAs have been reported in a few patients with EGPA but are much more prevalent in GPA. For this reason, its presence works against a patient with small vessel vasculitis being classified as EGPA. It is noteworthy that, in this case, the patient did not present with anti-PR3-ANCA, so this finding would be suggestive of EGPA over other small vessel vasculitides. Therefore, it counts as a positive criterion. Regarding the presence of anti-MPO-ANCAs, although they can be detected in EGPA patients, they are not useful as discriminant classifiers as they are more prevalent in other forms of vasculitis, such as microscopic polyangiitis. For this reason, they were not included in the final criteria [7]. Therefore, supporting this diagnosis is the good response to the introduction of corticoid therapy, the presence of isolated ANCA-MPO antibodies, whose presence, although not diagnostic, occurs in 30%-60% of patients [11], and finally, the absence of anti-PR3-ANCA. Despite the possibility for neurological involvement (mononeuritis multiplex), cutaneous vasculitis lesions, and hematuria, not found in this patient, pulmonary symptoms prevailed.

## Conclusions

It is important to point out the long diagnostic delay in the geriatric patient, much older than the median age of diagnosis for EGPA. This patient presented previously with a known diagnosis of chronic obstructive pulmonary disease (COPD), severe chronic rhinosinusitis with nasal polyposis, and cardiac diastolic dysfunction, culminating in a curious case of uncommon pleuroparenchymal involvement. Therefore, it is a pivotal example of the necessity of a broad and extensive clinical assessment in older patients in the setting of unexplained chronic multisystemic disease. Laboratory and imaging findings need to be interpreted in lieu of the patient’s medical history, as was important in this case in which the initial diagnosis of CAP was revealed incorrect, thus changing the therapeutic approach and prognosis.
